# Antitrypanosomal therapy for Chagas disease: A single center experience with adverse drug reactions and strategies for enhancing treatment completion

**DOI:** 10.1371/journal.pntd.0013218

**Published:** 2025-07-07

**Authors:** Katherine Reifler, Alyse Wheelock, Samantha M. Hall, Madolyn Dauphinais, Samuel Roytburd, Michael Maiullari, Alejandra Salazar, Ashley Maldonado, Helen Mahoney West, Julia R. Köhler, Elizabeth D. Barnett, Deepa M. Gopal, Davidson H. Hamer, Daniel L. Bourque

**Affiliations:** 1 Section of Infectious Disease, Department of Medicine, Boston University Chobanian & Avedisian School of Medicine, Boston, Massachusetts, United States of America; 2 Section of Preventative Medicine and Epidemiology, Boston University Chobanian & Avedisian School of Medicine, Boston, Massachusetts, United States of America; 3 Department of Environmental Health, Boston University School of Public Health, Boston, Massachusetts, United States of America; 4 Boston University Chobanian and Avedisian School of Medicine, Boston, Massachusetts, United States of America; 5 Division of Infectious Disease, Boston Children’s Hospital, Boston, Massachusetts, United States of America; 6 Harvard Medical School, Boston, Massachusetts, United States of America; 7 Section of Pediatric Infectious Disease, Department of Pediatrics, Boston Medical Center and Boston University Chobanian & Avedisian School of Medicine, Boston, Massachusetts, United States of America; 8 Cardiovascular Division, Department of Medicine, Boston University Chobanian & Avedisian School of Medicine, Boston, Massachusetts, United States of America; 9 Department of Global Health, Boston University School of Public Health, Boston, Massachusetts, United States of America; 10 Center for Emerging Infectious Disease Policy & Research, Boston University, Boston, Massachusetts, United States of America; Advanced Centre for Chronic and Rare Diseases, INDIA

## Abstract

Anti-trypanosomal therapy is generally recommended for individuals under age 50 with the indeterminate form of Chagas disease to prevent disease progression. However, benznidazole and nifurtimox are associated with adverse drug reactions. We performed a retrospective review of treatment tolerability among patients with Chagas disease referred to Boston Medical Center from June 2016 to June 2024. There were 125 individuals evaluated, of whom 32 (25.6%) had contraindications to and 2 (1.6%) declined antiparasitic treatment. Ninety-one started therapy (83 with benznidazole, 8 with nifurtimox) with monitoring co-managed by infectious diseases physicians and pharmacists. Following benznidazole initiation, 70 (84.3%) had at least one adverse event, of which allergic (39/83, 47.0%), gastrointestinal (38/83, 45.8%), and neuropsychiatric (33/83, 39.8%) reactions were most common. Rash led to treatment discontinuation in 19 patients (22.9%) and met criteria for grade 3 severity in 13 (15.7%). Adjunctive therapies for rash included topical and systemic steroids and systemic antihistamines. Peripheral neuropathy led to treatment cessation for 13 patients (15.7%). Gastrointestinal adverse effects occurred in 38 patients (45.8%), were relatively mild, and managed with H2 blockers or proton pump inhibitors. Thirty (36.1%) patients were unable to complete 60 days of benznidazole, of whom 15 switched to nifurtimox. Eight patients started with nifurtimox during a benznidazole shortage. Nifurtimox was more frequently associated with gastrointestinal side effects (21/23, 91.3%) compared to benznidazole. Ultimately, 83 patients (91.2%) received at least 30 days, and 68 patients (74.7%) completed at least 60 days of benznidazole or nifurtimox. Multiple strategies were used to prevent and alleviate adverse events; multi-disciplinary team management was essential. These findings underscore the support needed for individuals with Chagas disease to tolerate and complete therapy and highlight the need for safer and more effective options to facilitate access to treatment.

## Author summary

Chagas disease is caused by a parasitic infection most frequently occurring in Mexico, Central and South America. Approximately 300,000 individuals who migrated from these regions are living with Chagas disease in the US and may benefit from antiparasitic treatment. Yet many US providers are unfamiliar with treatment eligibility and management. Treatment with the two currently available medications (benznidazole and nifurtimox) is frequently associated with adverse drug reactions (ADRs) which can be challenging to manage. This study retrospectively described management of patients living with Chagas disease who were treated by a multidisciplinary team at Boston Medical Center. Most initiated treatment with benznidazole, but some were treated with nifurtimox due to benznidazole intolerance or shortage. Eighty-five percent of individuals who took benznidazole and 100% who took nifurtimox experienced ADRs. The most common benznidazole-associated ADR category was allergic, whereas for nifurtimox it was gastrointestinal. Despite significant ADRs, 75% completed the recommended 60 days of therapy, and 91% completed at least one month of therapy. This analysis underscores the significant side effect burden of currently available therapies for Chagas disease and that intensive treatment monitoring and support facilitates treatment completion. Tolerable and safe therapies are needed to expand access and improve treatment success.

## Introduction

Chagas disease (CD) is a neglected parasitic disease endemic to continental Latin America. Approximately 300,000 people are living with CD in the United States (US) [1]. Life-threatening cardiomyopathy or gastrointestinal sequelae develop in about 30% of people usually 10–30 years after initial infection with the parasite, *Trypanosoma cruzi* [2]. Although not entirely understood, this progression to advanced disease is thought to be triggered by persistent parasite infection in addition to immune-mediated tissue damage [3,4]. There are at least six different discrete type units (DTU) of *T. cruzi* which vary across geographic regions and along with host factors may explain the different manifestations of infection [5,6].

Two trypanocidal medications, benznidazole and nifurtimox, are used for treatment of Chagas disease and are available in the US [7,8]. Although the exact mechanisms of action are unknown, both are pro-drugs activated by the parasite’s mitochondrial nitroreductase to create metabolites that damage the parasite’s genomic DNA [9]. International and domestic guidelines [2018 American Heart Association (AHA), the Centers for Disease Control and Prevention, and 2019 Pan American Health Organization (PAHO)] do not state a preferred treatment regimen, but benznidazole is preferred by most experts as first-line therapy [10–12]. Treatment with benznidazole or nifurtimox prevents congenital transmission [13–15], is associated with high parasite clearance rates for acute [16], congenital [17], and early chronic infection in children [18], and may minimize disease progression in adults with chronic indeterminate (without organ damage) infection [19–22]. Antitrypanosomal therapy is recommended for these individuals given some evidence indicating it may prevent disease progression and improve mortality [10,11,19,23]. Despite potential eligibility for treatment, it is estimated that less than 1% of people living with CD in the US receive antiparasitic treatment [24].

Several barriers to treatment persist in non-endemic countries including cost and access to the medications [25], availability of specialist physicians and medication tolerability [26–28]. Adverse drug reactions (ADRs) to benznidazole and nifurtimox are common, particularly in adults [4,8,29–32]. The most common ADRs noted with benznidazole include rash, headache, anorexia, and irreversible neuropathy [30]. The most common ADRs observed with nifurtimox include anorexia, nausea, headache, and fatigue [4]. ADRs can be severe and lead to treatment termination; in a review of 30 patients treated in the US with benznidazole, all 30 had ADRs including 12 that were categorized as severe, though none were listed as life-threatening [31]. In a review of 176 patients treated 2008–2016 at a single site in Switzerland, at least one ADR was observed in 89.8% and ADRs predicted early treatment termination [29]. Despite the known high incidence of antitrypanosomal ADRs, guidance for clinicians for managing ADRs are limited.

In this study we aimed to 1) describe the incidence and severity of ADRs to benznidazole and nifurtimox in a cohort of individuals living in the Boston area who are predominantly from Central America; 2) determine risk factors associated with incomplete treatment; and 3) describe management and ADR mitigating factors among those who experienced ADRs and completed treatment. We hypothesize that a multidisciplinary management team approach to guide patients through Chagas disease therapy would improve treatment completion rates.

## Methods

### Ethics statement

The study data were collected as secondary data and are presented in aggregate without any identifying information and individual consent was not obtained. Research approval was obtained by the Institutional Review Board of BMC and community board approval from East Boston Neighborhood Health Center (H-39646) to view health center laboratory results.

### Study setting, protocol, and data collection

We conducted a single-site retrospective analysis of adult patients aged 18 years or older diagnosed with Chagas disease. Individuals included in the study underwent evaluation for antitrypanosomal therapy between January 2016 and June 2024 at the Center for Infectious Diseases (CID) at Boston Medical Center (BMC), a large academic safety net hospital located in Boston, Massachusetts. Most patients were identified through Chagas disease screening via the Strong Hearts Project at the East Boston Neighborhood Health Center [33].

Individuals were considered to have a diagnosis of Chagas disease if they had at least two positive serologic assays with distinct assays using different antigens. After an initial evaluation which included baseline blood chemistries, complete blood count, electrocardiogram (ECG), and transthoracic echocardiogram (TTE) with contrast, patients were staged using the American Heart Association (AMA) classification of Chagas disease (see S1 Table), according to standard clinical practice [34,35]. Screening for strongyloidiasis and latent tuberculosis was also done given the possibility of corticosteroid exposure during antitrypanosomal therapy and the risk of disseminated strongyloidiasis or tuberculosis reactivation [36–38]. Individuals with dyspepsia or epigastric pain were tested for *Helicobacter pylori* infection and, if positive, were treated prior to antitrypanosomal therapy. Chart review of infectious disease pharmacists’ and physicians’ notes in the electronic medical record was performed through September 2024 for demographic data, medical comorbidities, and treatment course, including medication dosing, duration of therapy, and ADRs.

Benznidazole was recommended as first-line therapy for adults ages 18–50 in the indeterminate stage without advanced heart disease, according to expert guidelines [10,11,39]. Nifurtimox was considered second-line therapy given potential for greater rates of ADRs and higher pill burden [12]. Treatment was considered for some patients over age 50 and for some individuals with evidence of early Chagas cardiomyopathy through shared decision-making with the patient and discussion with a heart failure specialist [10,40]. Pregnant people and patients with multiple medical comorbidities, at the discretion of the infectious disease clinician, were not offered antitrypanosomal therapy given toxicity concerns.

Initially, the benznidazole dosing was based on the Center for Disease Control and Prevention (CDC) recommendation to give 5 mg/kg/day up to 300 mg/day for 60 days—with extension past 60 days to complete the equivalent dose [2]. In 2019, the treatment approach changed to cap the dose at 300 mg daily for 60 days, regardless of the patient’s weight based on CDC and international guidelines [10]. Patients with benznidazole intolerance were switched to nifurtimox 8–10 mg/kg/day. Nifurtimox duration was initially set at 90 days until 2021, when the therapy duration was reduced based on a pediatric study that demonstrated acceptable seroconversion and seroreduction with a 60 day course of treatment [10,12,41,42]. In more recent years, there was a lower threshold to stop treatment between 30–60 days if ADRs developed, given mounting evidence supporting shorter treatment durations [43,44]. Both benznidazole and nifurtimox have been available since gaining FDA approval (benznidazole in 2017 for children aged 2–12 years old; nifurtimox from birth to age 18 in 2020). Prior to their approvals, they were obtained via the CDC investigational drug protocol [7,8].

While on treatment, follow-up appointments were scheduled every two weeks with an infectious disease pharmacist and monthly with an infectious disease physician. At each follow-up appointment, patients were asked about the occurrence of any benznidazole or nifurtimox ADRs. Specific ADRs were managed according to their severity and at the clinician’s discretion. Mild and moderate ADRs were initially managed medically. For example, anti-histamine-1 blockers, topical steroids, and/or oral steroids for itching or rash, H2 blockers or proton pump inhibitors for dyspepsia, antiemetics for nausea or anorexia, and analgesics for headache. Treatment was suspended temporarily (for days to weeks) when an ADR failed to improve with medical management. Antitrypanosomal therapy then was restarted at a low dose and gradually increased until reaching the target dose as tolerated [45]. Therapy was stopped for severe or potentially irreversible reactions, such as angioedema or peripheral neuropathy.

### Study definitions

Treatment duration was defined as days on target dose of antitrypanosomal therapy. The target dose and durations were defined above. Days of missed and/or below target dose of therapy were not counted for patients receiving the dose escalation strategy. ADR was defined as any symptom that started or worsened after initiation of the antitrypanosomal medication without a clear alternative explanation. The Common Terminology Criteria for Adverse Events (CTCAE v5.0) was used to determine ADR severity (1 = mild, 2 = moderate, 3 = severe, 4 = life-threatening, 5 = death) [46]. ADRs were grouped into allergic, gastrointestinal, neuropsychiatric, and systemic symptoms.

### Analysis

Descriptive analysis was stratified by antiparasitic treatment duration and type of antiparasitic therapy received to evaluate frequency, timing, and severity of ADRs. Standard deviation (SD) was calculated for the mean age of patients in the study. Chi-square analysis was used to assess any statistically significant difference in the proportion of ADR experience by treatment type (benznidazole vs nifurtimox), and to evaluate differences in demographic and clinical characteristics across those who did and did not complete therapy. Analyses were conducted in Rstudio (version 12.1; RStudio Team, 2024).

## Results

**Fig 1:**
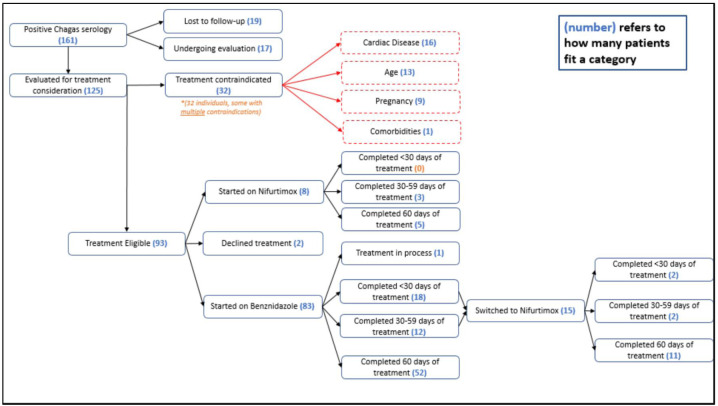
Antitrypanosomal Therapy Care Cascade.

Of the 161 patients diagnosed with Chagas disease, 36 were excluded from analysis due to loss to follow-up (n = 19) or incomplete evaluation at the time of analysis (n = 17) [S2 Table] and 125 completed evaluation for antiparasitic treatment [Fig 1]. Approximately 26% (32/125) were considered to have contraindications to therapy, including cardiac disease (16/32, 50%), advanced age (13/32, 41%), pregnancy (9/32, 28%), and/or other comorbidities (1/32, 3%); some had multiple contraindications. Most individuals (93/125, 74%) were considered eligible for antiparasitic therapy and 98% (91/93) of eligible individuals agreed to treatment. Two declined therapy.

### Cohort characteristics

Among those individuals who were candidates for treatment, 67% (84/125) were female. Most individuals migrated from Central America (95%, 119), a minority from South America (5%, 6), and none from Mexico; females comprised 67% (84/125) [Table 1]. The mean age was 44 years (SD 13). Additional comorbidities in this cohort included diabetes mellitus (15%, 19/125), coronary artery disease (12%, 15/125), chronic kidney disease (3%, 4/125), baseline liver dysfunction (6%, 7/125), strongyloidiasis seropositivity (14%, 17/125), and latent tuberculosis (14%, 18/125). Among individuals with possible symptoms of *H. pylori* infection who were tested, 47% (59/84) were positive and all received *H. pylori* treatment prior to antiparasitic therapy initiation.

**Table 1:** Chagas Disease Cohort Baseline Characteristics, Stratified by Antitrypanosomal Therapy Initiation and Therapy Completion Status.

	Total completed evaluation for anti-parasitic therapy (N = 125)	Treatment contra-indicated (N = 32)	Treatment eligible (N = 93)				
			Initiated treatment (N = 91)				Declined treatment, N = 2
			Initiated treatment total (N = 91)	Incomplete therapy <30 days (BNZ or NFX) N = 7	Incomplete therapy (30–59 days BNZ or NFX) N = 15	Complete therapy (at least 60 days BNZ or NFX) N = 68	
**Female, n (%)**	84 (67.2%)	22 (68.8%)	60 (65.9%)	5/7 (71.4%)	10 (66.7%)	44 (64.7%)	2 (100%)
**Country of birth, n (%)**							
Central America	119 (95.2%)	29 (90.6%)	88 (96.7%)	7 (100%)	15 (100%)	65 (95.6%)	2 (100%)
South America	6 (4.8%)	3 (9.4%)	3 (3.3%)			3 (4.4%)	
**Age (years), mean (SD)**	44 (13)	52 (17)	42 (11)	41 (8)	40 (8)	42 (12)	54 (0)
**AHA cardiomyopathy stage, n (%)**							
A	64 (51.2%)	8 (25.0%)	54 (59.3%)	5 (71.4%)	9 (60.0%)	40 (58.8%)	2 (100%)
B1	37 (29.6%)	5 (15.6%)	32 (35.2%)	2 (28.6%)	4 (26.7%)	25 (36.8%)	
B2	9 (7.2%)	4 (12.5%)	5 (5.5%)		2 (13.3%)	3 (4.4%)	
C	12 (9.6%)	12 (37.5%)					
D	1 (0.8%)	1 (3.1%)					
Missing	2 (1.6%)	2 (6.3%)					
**Diabetes mellitus status, n (%)**							
Diabetes mellitus	19 (15.2%)	9 (28.1%)	8 (8.8%)	1 (14.3%)	1 (6.7%)	5 (7.3%)	2 (100%)
Missing	18 (14.4%)	5 (15.6%)					
**CAD status, n (%)**							
CAD	15 (12%)	13 (40.6%)	2 (2.2%)			2 (2.9%)	
Missing	7 (5.6%)	2 (6.3%)			1 (6.7%)		1 (50%)
**CKD status, n (%)**							
CKD	4 (3.2%)	4 (12.5%)					
Missing	3 (2.4%)	3 (9.4%)					
**Baseline liver dysfunction status, n (%)**							
Liver dysfunction	7 (5.6%)	2 (6.3%)	5 (5.5%)		1 (6.7%)	4 (5.9%)	
Missing	8 (6.4%)	5 (15.6%)					
*Helicobacter pylori* status, n (%)**							
*H. pylori* positive	59/84 (47.2%)	9/17 (52.9%)	48/65 (73.8%)	3/3 (100%)	9/11 (81.8%)	36/50 (72%)	2/2 (100%)
**Strongyloidiasis status, n (%)**							
*Strongyloides stercoralis* IgG seropositive	17 (13.6%)	3 (9.4%)	14 (15.4%)		2 (13.3%)	12 (17.6%)	
Missing	19 (15.2%)	10 (3.1%)		1 (14.3%)			
**QuantiFERON gold status, n (%)**							
QuantiFERON gold positive	18 (14.4%)	4 (12.5%)	14 (15.4%)	1 (14.3%)	1 (6.7%)	12 (17.6%)	
Missing	19 (15.2%)	10 (3.1%)					

BNZ = benznidazole; NFX = Nifurtimox; AHA = American Heart Association; CAD = coronary artery disease; CKD = chronic kidney disease; *H. pylori* = *helicobacter pylori*.

**H. pylori only sent in those with concerning symptoms.

With respect to cardiac status, more than half had no evidence of cardiomyopathy (51%, 64/125 AHA stage A), approximately one-third had evidence of early cardiac changes (30%, 37/125 AHA stage B1), and 18% (22/125) had evidence of Chagas cardiomyopathy stage B2 or higher [Table 1]. Among 91 individuals who initiated therapy, 54 (59%) were stage A, 32 (35%) were stage B1, and 5 (6%) were stage B2. No stage C or D individual initiated therapy.

### Antiparasitic therapy completion

Among 91 patients who started therapy, most (83, 91%) initiated first-line benznidazole [Fig 1]. However, this study included a period of benznidazole shortage in the US during which nifurtimox was started as an alternative therapy for 8 individuals (9%). Similar proportions completed ≥ 60 days of the first drug prescribed: 61% (51/83) with benznidazole and 63% (5/8) with nifurtimox. Many were unable to tolerate 60 days of therapy; 23% (18/83) who started benznidazole completed <30 days of therapy, 14% (12/83) completed between 30–59 days, and one individual was still receiving treatment at the time of analysis. Amongst 30 individuals who could not complete 60 days of benznidazole, half were switched to nifurtimox and 11 of 15 completed 60 days of nifurtimox. The remaining 4 individuals could not complete therapy with either benznidazole or nifurtimox. No patients that were initiated on nifurtimox treatment for <60 days were switched to benznidazole. Overall, 83 patients (91%) received at least one month, and 68 patients (75%) completed at least 60 days of either benznidazole or nifurtimox [Table 1].

### Adverse drug reactions

ADRs were common, occurring in 84% (70/83) who received benznidazole and 100% (23/23) of those who received nifurtimox [Table 2]. Amongst 83 individuals who received benznidazole, allergic ADRs were most common (39, 47%). Rash, the most common allergic benznidazole ADR subtype (36/83, 43%), was severe for 13 (16%) individuals and led to treatment discontinuation in 19 (23%). Two people developed angioedema and one developed drug reaction with eosinophilia and systemic symptoms (DRESS). Five individuals had a non-severe transaminase rise, and all returned to baseline with medication pause. Overall, benznidazole gastrointestinal ADRs were less severe and less likely to necessitate treatment discontinuation. The most common neuropsychiatric benznidazole ADR subtype, peripheral neuropathy, occurred in 17 patients (21%) and led to treatment discontinuation in 13 (16%). No individual developed peripheral neuropathy categorized as severe. For the 4 individuals who did not stop therapy due to peripheral neuropathy, reasons included the patient notifying the pharmacist about their ADR after therapy completion (n = 2) and the symptom initially not thought to be neuropathy (n = 2).

**Table 2:** Adverse Drug Reactions Associated with Benznidazole and Nifurtimox Therapy.

Adverse Drug Reaction	Benznidazole (N = 83), n(%)					Nifurtimox (N = 23), n (%)					Proportion of ADRs associated with BNZ vs NFX (p-value)
	Total	ADR leading to treatment disconti-nuation	Severity, n (%)			Total	ADR leading to treatment disconti-nuation	Severity, n (%)			
			Mild (grade 1)	Moderate (grade 2)	Severe or worse (grade ≥3)			Mild (grade 1)	Moderate (grade 2)	Severe or worse (grade ≥3)	
**Any AE**	70/83 (84.3%)					23/23 (100%)					0.095
**Allergic**	39/83 (47%)					10/23 (43.5%)					0.950
Rash	36/83 (43.4%)	19/36 (52.8%)	4/36 (11.1%)	19/36 (52.8%)	13/36 (36.1%)	6/23 (26.1%)	2/6 (33.3%)	3/6 (50.0%)	1/6 (16.7%)	2/6 (33.3%)	
Pruritis	7/83 (8.4%)	1/7 (14.3%)	2/7 (28.6%)	4/7 (57.1%)	1/7 (14.2%)	4/23 (17.4%)	1/4 (25.0%)	3/4 (75.0%)	1/4 (25.0%)		
Angioedema	2/83 (2.4%)	2/2 (100%)			2/2 (100%)	1/23 (4.3%)	1/1 (100%)			1/1 (100%)	
DRESS	1/83 (1.2%)	1/1 (100%)		1/1 (100%)							
**Gastrointestinal**	38/83 (45.8%)					21/23 (91.3%)					**0.0003**
Dyspepsia/abdominal pain	15/83 (18.1%)	1/15 (6.7%)	12/15 (80.0%)	3/15 (20.0%)		9/23 (39.1%)	2/9 (22.2%)	5/9 (55.6%)	3/9 (33.3%)	1/9 (11.1%)	
Nausea	11/83 (13.3%)	1/11 (9.1%)	7/11 (63.6%)	4/11 (36.4%)		9/23 (39.1%)	2/9 (22.2%)	4/9 (44.4%)	4/9 (44.4%)	1/9 (11.1%)	
Heartburn	10/83 (12.0%)	1/10 (10.0%)	5/10 (50.0%)	5/10 (50.0%)		7/23 (30.4%)	1/7 (14.3%)	1/7 (14.3%)	6/7 (86.7%)		
Anorexia/decreased appetite	8/83 (9.6%)		7/8 (87.5%)	1/8 (12.5%)		7/23 (30.4%)	1/7 (14.3%)	4/7 (57.1%)	3/7 (42.9%)		
Bloating	7/83 (8.4%)		6/7 (85.7%)	1/7 (14.2%)							
Diarrhea	3/83 (3.6%)		2/3 (66.7%)	1/3 (33.3%)							
Dysgeusia	1/83 (1.2%)		1/1 (100%)			5/23 (21.7%)	1/5 (20.0%)	3/5 (60.0%)	2/5 (40.0%)		
Constipation	1/83 (1.2%)			1/1 (100%)							
Vomiting	1/83 (1.2%)			1/1 (100%)		3/23 (13.0%)	2/3 (66.7%)		2/3 (66.7%)	1/3 (33.3%)	
**Neuropsychiatric**	33/83 (39.8%)					15/23 (65.2%)					0.053
Peripheral neuropathy	17/83 (20.5%)	13/17 (76.5%)	7/17 (41.2%)	10/17 (58.8%)		8/23 (34.8%)	3/8 (37.5%)	5/8 (62.5%)	3/8 (37.5%)		
Headache	11/83 (13.3%)		10/11 (90.9%)	1/11 (9.1%)		7/23 (30.4%)	2/7 (28.6%)	5/7 (71.4%)	2/7 (28.6%)		
Dizziness/vertigo	6/83 (7.2%)		6/6 (100%)			3/23 (13.0%)	1/3 (33.3%)	1/3 (33.3%)	2/3 (66.7%)		
Insomnia	3/83 (3.6%)		1/3 (33.3%)	2/3 (66.7%)		4/23 (17.4%)	1/4 (25.0%)	1/4 (25.0%)	3/4 (75.0%)		
Anxiety						1/23 (4.3%)		1/1 (100%)			
**Systemic**	30/83 (36.1%)					7/23 (30.4%)					0.794
Fatigue	11/83 (13.3%)		11/11 (100%)			4/23 (17.4%)	1/4 (25.0%)	2/4 (50.0%)	2/4 (50.0%)		
Myalgias	6/83 (7.2%)		2/6 (33.3%)	3/6 (50.0%)	1/6 (16.7%)						
Arthralgias	6/83 (7.2%)		3/6 (50.0%)	3/6 (50.0%)					1/1 (100%)		
Transaminitis	5/83 (6.0%)	2/5 (40.0%)		3/5 (60.0%)	2/5 (40.0%)	1/23 (4.3%)		1/1 (100%)			
Hot flashes	5/83 (6.0%)		4/5 (80.0%)	1/5 (20.0%)							
Subjective fever	4/83 (4.8%)	1/4 (25.0%)	3/4 (75.0%)	1/4 (25.0%)		2/23 (8.7%)		1/2 (50%)	1/2 (50%)		
Erectile dysfunction	1/83 (1.2%)		1/1 (100%)								
Creatinine elevation	1/83 (1.2%)	1/1 (100%)	1/1 (100%)								
Palpitations						1/23 (4.3%)			1/1 (100%)		

Compared to benznidazole, nifurtimox was associated with more frequent gastrointestinal side effects (21/23 or 91% vs 36/83 or 43%; p-value 0.003). Two individuals had severe gastrointestinal ADRs to nifurtimox including multiple visits to the emergency department within one week for dyspepsia/abdominal pain or nausea and leading to treatment discontinuation. Neuropsychiatric ADRs were also more common in those treated with nifurtimox compared to benznidazole (15/23 or 65% vs 33/83 or 40%, p-value 0.053). Peripheral neuropathy was the most common neuropsychiatric nifurtimox ADR subtype and led to therapy discontinuation in 3 people, although no reaction was severe. Allergic nifurtimox ADRs were less frequent compared to benznidazole, although 3 individuals had severe allergic nifurtimox ADRs, including angioedema in one person.

### Adverse drug reaction timing

**Fig 2:**
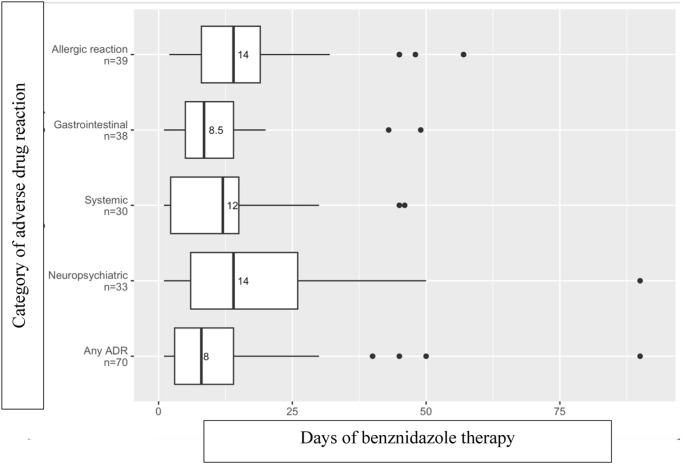
Days of Therapy to Onset of Adverse Drug Reactions Amongst 83 Individuals Treated with Benznidazole. Median shown by number and vertical line in box; interquartile range demonstrated by the box boundaries.

**Fig 3:**
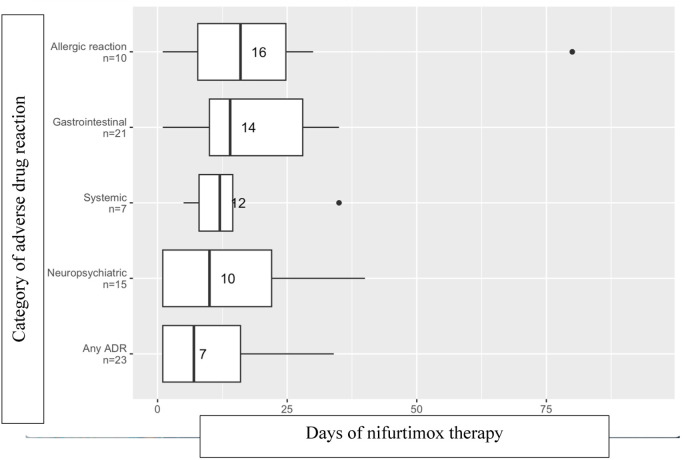
Days of Therapy to Onset of Adverse Drug Reactions Amongst 23 Individuals Treated with Nifurtimox. Median shown by number and vertical line in box; interquartile range demonstrated by the box boundaries.

Timing of initiation of therapy to ADR onset is shown in Figs 2 and 3. Most ADRs started within the first month of therapy. For benznidazole, gastrointestinal symptoms typically started earlier (median onset at 8.5 days), and allergic, systemic, and neuropsychiatric reactions (including peripheral neuropathy) typically started at approximately 2 weeks of therapy [Fig 2]. There was a wide range of ADR onset, particularly because this analysis included a period when the recommended dosing was cumulative weight-based without a duration cap, and some individuals with higher weights had > 60 days of therapy. The median onset of allergic and gastrointestinal ADRs for nifurtimox occurred a few days later (allergic: 16 days nifurtimox versus 14 days benznidazole; gastrointestinal: 14 days nifurtimox versus 8.5 days benznidazole), and those of neuropsychiatric ADRs occurred a few days earlier (10 days nifurtimox versus 14 days benznidazole) than for benznidazole. Median onset of systemic symptoms was similar (12 days for both nifurtimox and benznidazole). Similar to benznidazole, the median onset of nifurtimox ADRs overall started after approximately 2 weeks of therapy.

### Management of adverse drug reactions

Multiple strategies were used to prevent and alleviate adverse drug reactions and allow for treatment completion. Common ADRs and their management are presented in Table 3. Rash and pruritus were common allergic ADRs that were managed with oral antihistamine and/or oral or intravenous steroids, depending on severity. With this approach, 24/37 (65%) of individuals with rash and 7/11 (64%) of individuals with pruritus successfully completed 60 days of therapy. Gastrointestinal ADRs were managed with oral antihistamines if mild or proton pump inhibitors if severe, and 77% of individuals with this management completed therapy. Nausea was managed with serotonin receptor antagonists and bloating with simethicone; 68% and 86% of individuals with these ADRs, respectively, completed therapy. In patients with peripheral neuropathy, an ADR that may be irreversible, therapy was stopped and gabapentin or a topical analgesic added as needed. Forty-three percent (9/21) of individuals who developed peripheral neuropathy completed therapy, either because they did not notify their care team of the symptom until after therapy completion or by switching to second-line nifurtimox. Headaches, myalgias, and arthralgias were managed with over-the-counter acetaminophen or ibuprofen. Individuals who developed asymptomatic mild (grade 1) transaminase elevation compared to baseline were monitored with labs every 2 weeks, but those with moderate or severe (grade 2–3) stopped therapy. Half (3/6) of the individuals who developed transaminase elevation completed therapy safely with this approach.

**Table 3:** Management of Common Benznidazole and Nifurtimox Adverse Drug Reactions.

ADR category	ADR treatment	% Completed ≥ 60 days Chagas therapy
**Allergic**		
Rash		24/37 (64.9%)
Mild (grade 1)	Topical steroid + oral antihistamine	
Moderate (grade 2)	Topical steroid + oral antihistamine; oral steroid if not responsive	
Severe (grade 3)	Oral steroid; IV steroid if not responsive	
Pruritis		7/11 (63.6%)
Mild (grade 1)	Oral antihistamine	
Moderate (grade 2)	Topical steroid, oral antihistamine	
Severe (grade 3)	N/A	
**Gastrointestinal**		
Dyspepsia/abdominal pain/heartburn		26/34 (76.5%)
Mild (grade 1)	oral H2-blocker	
Moderate/severe (grade 2/3)	proton pump inhibitor	
Nausea		13/19 (68.4%)
Mild (grade 1)	no specific therapy	
Moderate/severe (grade 2/3)	5-HT3 receptor antagonist (i.e.,: ondansetron)	
Bloating grade 1–2)	simethicone	6/7 (85.7%)
**Neuropsychiatric**		
Peripheral neuropathy	Stop therapy; gabapentin and/or topical analgesic as needed	9/21 (42.9%)
Headache	acetaminophen or ibuprofen	12/18 (66.7%)
**Systemic**		
Myalgias/arthralgias	acetaminophen or ibuprofen	8/11 (72.7%)
Transaminitis		3/6 (50%)
Mild (grade 1)	Monitor labs every 2 weeks; consider pausing therapy and restarting with dose escalation protocol	
Moderate/severe grade 2/3)	stop therapy	

ADR = adverse drug reaction; H2-blocker = antihistamine-2 blocker; 5-HT3 receptor antagonist = serotonin receptor antagonist.

ADR grade defined according to CTCAE category.

### Factors associated with incomplete antiparasitic therapy

Several ADR factors were examined to assess their association with patients’ inability to complete therapy. Allergic ADRs (p 0.050), neuropsychiatric ADRs (p 0.050), any moderate ADR (grade 2 or worse, p 0.001), or any severe ADR (grade 3 or worse, p 0.012) were associated with an increased likelihood of incomplete treatment [Table 4]. A greater proportion of individuals with incomplete (86%) versus complete (67%) therapy experienced ≥ 2 ADRs, but this difference was not significant. Using a dose escalation protocol with initiation of antitrypanosomal treatment resulted in slightly fewer patients who did not complete therapy, though was not statistically significant (11/22 or 50% vs 37/68 or 54%; p 0.909).

**Table 4:** Factors Associated with Incomplete Treatment of Benznidazole or Nifurtimox <60 days.

	Incomplete treatment (n = 22)	Complete treatment (n = 68)	Difference in proportion of incomplete vs complete treatment ≥60 days (p-value)
**Demographic factors (n, %)**			
Female	15 (68.2%)	44 (64.5%)	0.968
Age > 50 years	2 (9.1%)	15 (22.1%)	1
**Medical history factors (n, %)**			
Baseline CKD	0	0	n/a
Baseline LFT abnormalities	1 (4.5%)	4 (5.9%)	1
Diabetes mellitus	2 (9.1%)	5 (7.4%)	1
CAD	0	2 (2.9%)	n/a
AHA CMY stage B1 (or higher)	8 (36.4%)	28 (41.2%)	0.881
**Medication factors (n, %)**			
Dose escalation*	11 (50.0%)	37 (54.4%)	0.909
BNZ Dose > 5 mg/kg/d	3 (13.6%)	8 (11.8%)	1
ADR factors** (n, %)			
Allergic ADR	15 (68.2%)	28 (41.2%)	**0.050**
Neuropsychiatric ADR	15 (68.2%)	28 (41.2%)	**0.050**
Gastrointestinal ADR	14 (63.6%)	38 (55.9%)	0.695
Systemic ADR	9 (40.9%)	25 (36.8%)	0.924
Any moderate ADR (grade 2 or worse)	20 (90.9%)	31 (45.6%)	**0.001**
Any severe ADR (grade 3 or worse)	8 (36.4%)	7 (10.3%)	**0.012**
≥ 2 ADRs	19 (86.4%)	45 (66.2%)	0.122

CKD = chronic kidney disease; LFT = liver function test; CAD = coronary artery disease; AHA = American Heart Association; CMY = cardiomyopathy; BNZ = benznidazole; mg/kg/d = milligram per kilogram per day; ADR = adverse drug reaction.

Fisher’s exact test used for values <5.

ADR grade defined according to CTCAE category.

*Individuals who had any dose escalation; if they had dose escalation for both benznidazole and nifurtimox, they are only counted once.

**Individuals who had a particular category of ADR to BNZ or NFX; if they had the same category of ADR to both BNZ & NFX are only counted once.

## Discussion

This cohort of 125 individuals, primarily from Central America, with Chagas disease assessed for treatment is one of the largest to date in the US to be followed for management of ADRs to benznidazole and nifurtimox. Approximately three-quarters were considered eligible for antiparasitic treatment and almost every patient who was offered treatment elected to start. More than half were female, perhaps due to more consistent access to health care and screening during pregnancy. ADRs were common, occurring in 84% (70/83) of those who received benznidazole and in 100% (23/23) of those who received nifurtimox. Several patients experienced treatment-limiting ADRs, including most frequently rash and peripheral neuropathy, and some experienced severe ADRs including rash, angioedema, and DRESS. In some cases, the ADRs had additional negative outcomes not necessarily captured by the medical grading system that are particularly disadvantageous in this already vulnerable population, including inability to work or provide childcare during the ADR. Our ADR findings fit within the range of prior studies in endemic and non-endemic regions [47–50].

Several studies have examined whether the effective antiparasitic dose may be lower, as this strategy might minimize ADRs. The BENDITA double-blinded multicenter randomized control trial suggested that therapy duration as short as 14 days compared to the current 60-day standard is associated with similar rates of sustained parasite clearance at 6 months [43]. MULTIBENZ, another recent double-blinded multicenter randomized control trial, demonstrated that lower treatment dose (benznidazole 150 mg/kg/d compared to the current standard of 300 mg/kg/d) for 60 days is associated with similar parasitological clearance at 12 months [44]. Given most ADRs in our cohort started within the first month of therapy, a 30-day therapy duration or lower dose therapy may not avoid ADRs, but perhaps could minimize ADR duration and improve treatment completion rates. The timing of ADR onset in the BENDITA and MULTIBENZ trials aligns with our cohort, with most ADRs occurring within the first 30 days of therapy [43,44]. While these studies show parasite clearance with shorter therapy durations, the impact of a shorter duration and/or dose of antiparasitic therapy on long-term outcomes of people with Chagas disease is unknown.

Despite high rates of ADRs, 91% of patients in our cohort received at least 30 days, and 75% completed at least 60 days of either benznidazole or nifurtimox. At 30 days, treatment completion in our cohort was higher than a pooled completion rate of 14% reported in a systematic review [51]. However, at 60 days, treatment completion in our cohort was similar to, or slightly lower than, treatment completion in other cohorts [32]. We utilized several methods to prevent and mitigate ADRs and improve therapy completion, including dose-escalation, a multidisciplinary care approach that involved frequent follow up with pharmacists and physicians, medical management of ADRs, and, for some individuals, switching therapies. We treated *H. pylori* positive individuals with relevant gastrointestinal complaints prior to starting benznidazole or nifurtimox, a strategy that may decrease gastrointestinal ADRs. We screened for strongyloidiasis and treated with ivermectin if seropositive to minimize potential consequences of oral corticosteroids used to mitigate allergic ADRs. In recent years we initiated therapy with dose-escalation for all patients based on findings in Spain that this approach may improve benznidazole treatment completion at 60 days [52]. This study was not designed to assess the dose escalation strategy, but the proportions of individuals who received the dose-escalation strategy in our cohort who did and did not complete therapy were similar. Prospective studies are needed to confirm the efficacy of this approach [53]. Other methods of potentially mitigating ADRs proposed by other groups include testing for the HLA-B35:05 allele, whose presence may be associated with risk of benznidazole-associated dermatitis, and co-administration of L-ascorbic acid with benznidazole, suggesting that oxidative stress plays a significant role in the mechanism of benznidazole toxicity [54,55]. These strategies are experimental and further research to examine and validate factors associated with antitrypanosomal toxicity is needed.

Strengths of this analysis include a well-characterized cohort in a non-endemic setting with excellent follow-up and frequent monitoring, enabling us to understand the spectrum of ADRs with both Chagas disease therapies that are commercially available in the US. Limitations include the relatively small sample size and inability to perform formal power or multivariable regression analysis, and the imbalance between the number of individuals treated with benznidazole versus nifurtimox. However, most centers that manage large numbers of individuals with Chagas disease start with benznidazole and only change to nifurtimox if the former drug is poorly tolerated [11,56]. The single-center experience with a primarily Central American cohort may limit generalizability to other non-endemic settings with different health systems and/or more South Americans. Whether treatment ADRs differ between Central and South Americans is not well studied. Another limitation is that the treatment approach was not uniform across the entire cohort because the recommended dosing and medication availability changed throughout the study period. In addition, our approaches to mitigating adverse effects evolved over the course of this study period. Nonetheless, it is unlikely these factors had a significant impact on our findings.

This study underscores the fact that adverse drug events continue to pose major barriers to successful antitrypanosomal treatment and require significant resources to manage. We demonstrate that ADRs were often treatment-limiting, despite frequent monitoring along with ADR mitigation strategies, availability of treatment, and experienced providers. We outline strategies to manage common ADRs, aiming to provide guidance for other providers in their approach to the management of antitrypanosomal therapy. Ultimately, research into mechanisms behind drug toxicity, alternative therapies, and multi-center randomized control trials with long-terms outcomes comparing different dosing strategies are needed. Addressing these issues will require concerted efforts in drug development and dedication to high-quality clinical trials to reduce the burden of this neglected disease and improve patient outcomes.

## Suppporting information

S1 Table American Heart Association Classification of Chagas Cardiomyopathy. Arrhythmias and conduction disease can occur from B1 through D stages. HF, heart failure; NYHA, New York Heart Association. Adapted from Andrade et al. [55]. This is an Open Access article distributed under the terms of the Creative Commons Attribution License, which permits unrestricted use, distribution, and reproduction in any medium, provided the original author and source are credited.
(DOCX)

S2 Table Characteristics of Individuals Lost to Follow Up and Incompletely Evaluated for Antitrypanosomal Therapy. AHA, American Heart Association.
(DOCX)
