# Supplementary material for: Antitrypanosomal therapy for Chagas disease: A single center experience with adverse drug reactions and strategies for enhancing treatment completion
Source: PLoS Negl Trop Dis. 2025 Jul 7;19(7):e0013218. doi: 10.1371/journal.pntd.0013218 (PMC12233308; doi:10.1371/journal.pntd.0013218)
Supplement: S1 Table — Arrhythmias and conduction disease can occur from B1 through D stages. HF, heart failure; NYHA, New York Heart Association. Adapted from Andrade et al. [55]. This is an Open Access article distributed under the terms of the Creative Commons Attribution License, which permits unrestricted use, distribution, and reproduction in any medium, provided the original author and source are credited. (DOCX) [file pntd.0013218.s001.docx]

| **Definitions and Progression of Chagas Disease** | | | | |  |
| --- | --- | --- | --- | --- | --- |
| ***Indeterminate Form*** | ***Chagas Cardiomyopathy*** | | | |  |
| A | B1 | Chagas Dilated Cardiomyopathy/Heart Failure | | |  |
| Patients at risk for developing heart failure. They have positive serology, neither structural cardiopathy nor HF symptoms. Normal ECG. | Patients with structural cardiomyopathy, evidenced by ECG or TTE changes, but with normal global ventricular function and neither current nor previous signs or symptoms of HF. | B2 | C | D |  |
|  |  | Patients with structural cardiomyopathy characterized by global ventricular dysfunction and neither current nor previous signs and symptoms of HF. | Patients with ventricular dysfunction and current or previous symptoms of HF  (NYHA functional class I, II, III, or IV). | Patients with refractory symptoms of HF at rest despite optimized clinical treatment requiring special interventions |  |
|  |  |  |  |  |  |
|  |  |  |  |  |  |
|  |  |  |  |  |  |
|  |  |  |  |  |  |
|  |  |  |  |  |  |
|  |  |  |  |  |  |
|  |  |  |  |  |  |
|  |  |  |  |  |  |
|  |  |  |  |  |  |
|  |  |  |  |  |  |
|  |  |  |  |  |  |
|  |  |  |  |  |  |

S1 Table: American Heart Association Classification of Chagas Cardiomyopathy
