# Supplementary material for: Antitrypanosomal therapy for Chagas disease: A single center experience with adverse drug reactions and strategies for enhancing treatment completion
Source: PLoS Negl Trop Dis. 2025 Jul 7;19(7):e0013218. doi: 10.1371/journal.pntd.0013218 (PMC12233308; doi:10.1371/journal.pntd.0013218)
Supplement: S2 Table — AHA, American Heart Association. (DOCX) [file pntd.0013218.s002.docx]

**S2 Table: Characteristics of Individuals Lost to Follow Up and Incompletely Evaluated for Antitrypanosomal Therapy**

|  | Lost to Follow-Up, **N=19** | Undergoing evaluation, **N=17** |  |
| --- | --- | --- | --- |
|  |  |  |  |
|  |  |  |  |
| **Female, n (%)** | 6/19 (31.6%) | 7/17 (41.2%) |  |
| **Age (years), mean [SD]** | 42 [12] | 44 [11] |  |
| **AHA cardiomyopathy stage, n (%)** |  |  |  |
| A | 7/19 (36.8%) | 4/17 (23.5%) |  |
| B1 | 2/19 (10.5%) | 4/17 (23.5%) |  |
| B2 | 1/19 (5.3%) | 1/17 (5.9%) |  |
| C | 1/19 (5.3%) | 0 |  |
| D | 0 | 0 |  |
| Missing | 8/19 (42.1%) | 6/17 (35.3%) |  |
| **Diabetes mellitus status, n(%)** |  |  |  |
| Diabetes | 1/19 (5.3%) | 2/17 (11.8%) |  |
| Missing | 4/19 (21.1%) | 7/17 (41.2%) |  |
| **Coronary artery disease, n(%)** |  |  |  |
| CAD | 1/19 (5.3%) | 0 |  |
| Missing | 8/19 (42.1%) | 10/17 (58.8%) |  |
| **Chronic kidney disease, n(%)** |  |  |  |
| CKD | 0 | 1/17 (5.9%) |  |
| Missing | 3/19 (15.8%) | 4/17 (23.5%) |  |
| **Baseline liver dysfunction, n (%)** |  |  |  |
| Liver dysfunction | 0 | 1/17 (5.9%) |  |
| Missing | 4/19 (21.1%) | 4/17 (23.5%) |  |
| ***H. pylori* status, n(%)** |  |  |  |
| *Helicobacter pylori* + | 5/19 (26.3%) | 5/17 (29.4%) |  |
| Missing | 13/19 (68.4%) | 9/17 (52.9%) |  |
| **Strongyloidiasis status, n (%)** |  |  |  |
| *Strongyloides stercoralis* IgG+ | 0 | 1/17 (5.9%) |  |
| Missing | 12/19 (63.2%) | 8/17 (47.1%) |  |
| **QuantiFERON Gold status, n (%)** |  |  |  |
| QuantiFERON Gold+ | 4/19 (21.1%) | 0 |  |
| Missing | 6/19 (31.6%) | 5/17 (29.4%) |  |
